# A novel sensitive analytical method for the simultaneous analysis of vancomycin and teicoplanin in human urine via single high‐performance liquid chromatography coupled with photodiode array and mass spectrometry in series

**DOI:** 10.1002/jssc.202200002

**Published:** 2022-05-20

**Authors:** Maria Giakoumaki, Yiannis Sarigiannis, Evroula Hapeshi

**Affiliations:** ^1^ Department of Life and Health Sciences School of Sciences and Engineering University of Nicosia Nicosia Cyprus

**Keywords:** antibiotics analysis, human urine, solid‐phase extraction, teicoplanin, vancomycin

## Abstract

Analysis of vancomycin and teicoplanin in biological fluids is vital since they are used in the treatment of hospital infections. For the determination of both glycopeptides in urine, a sensitive and accurate analytical method using high‐performance liquid chromatography coupled with photodiode array and mass spectrometry was developed and validated. This research work is the first attempt to develop a chromatographic method for the determination of two glycopeptides with structural similarities. Moreover, the used non‐invasive sampling method is an advantage of this research effort, especially when the blood sampling is difficult. Urine was treated with acetonitrile and 5% trichloroacetic acid, followed by solid‐phase extraction. The chromatographic separation was established at a C18 column (4.6 × 150 mm, 5 μm), using a gradient method and an electrospray ionization source in a positive mode. The linearity of the method was *R* ^2^≥ 0.9900. The precision was estimated with a maximum coefficient of variation below 15%, while the accuracy ranged from 64 to 121%. The limit of detection and quantification of both glycopeptides ranged from 0.076 up to 0.33 mg/L and 0.33 up to 2.1 mg/L, respectively, showing the same sensitivity as the triple quadrupole mass spectrometry, which is the most frequently used method.

**Article Related Abbreviations**
CV: coefficient of variation
DAD: diode array detector
EMA: European Medicines Agency
FA: formic acid
MeOH: methanol
PDA: photodiode array detector
PP: protein precipitation
SIR: selected ion recording
TCA: trichloroacetic acid
TEIC: teicoplanin
VANC: vancomycin

## 1 INTRODUCTION

The glycopeptides, vancomycin (VANC), and teicoplanin (TEIC) are mainly utilized as a shield against Gram‐positive bacteria such as *Staphylococcus aureus, Enterococcus* spp.*, Clostridium difficile*. They are considered the priority substances in the treatment of hospital infections by resistant bacteria like methicillin‐resistant *S. aureus* [1, 2]. Both VANC and TEIC are products of actinomyces metabolism. Glycopeptides inhibit the last steps in bacterial cell wall peptidoglycan biosynthesis [3]. They share the same glycosylated cyclic or polycyclic backbone of seven amino acids, showing only differences in some amino acids and in aromatic amino acid substituents which may be sugars or amino sugars [4].

**FIGURE 1:**
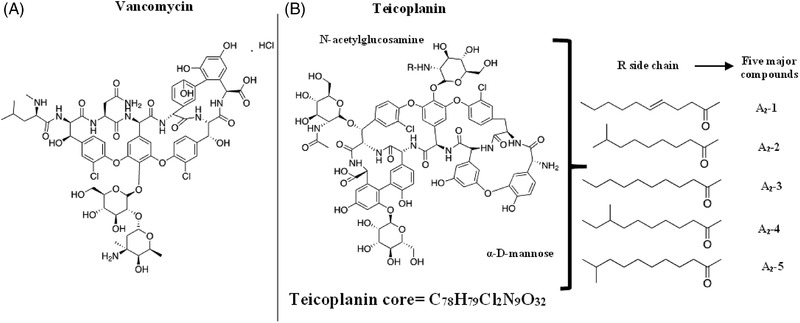
Structures of (A) vancomycin and (B) the major teicoplanin

Vancomycin (Figure 1) contains a glycosylated hexapeptide chain rich in amino acids, chloro‐β‐hydroxytyrosines, phydroxyphenylglycines, *N*‐methyl‐leucine, and aspartic acid [4]. Teicoplanin has as a major component TEIC A2 (90‐95%), which is a mixture of five other sub‐components (A2‐1, A2‐2, A2‐3, A2‐4, and A2‐5) with predominant A2‐2 and TEIC A3‐1 [2] (Figure 1). Teicoplanin can be classified into the lipoglycopeptide class due to the lipophilic side chain (R) of 10 to 11 carbon atoms which is linked to the amino sugar of TEIC [3].

According to the literature review, for the single determination of VANC and TEIC in biological samples such as urine, plasma, serum, and tissues, various analytical methods based on liquid chromatography have been developed. The RP‐HPLC coupled with an ultraviolet (UV) or diode array detector (DAD) was the predominant method for the analysis of these compounds [5, 6]. For example, Hu et al. developed a simple method using HPLC‐DAD with second‐order calibration algorithms to directly quantify VANC beyond other drugs in human plasma [5]. Several of the referred analytical methods based on HPLC coupled with different detection methods present both advantages and disadvantages. For example, RP‐HPLC with electrochemical or fluorescence detection were used as sensitive methods for the assessment of TEIC and VANC in various biological fluids [7]. Nevertheless, some drawbacks such as a long analysis time or the lower performance of the analysis due to the step of derivatization were presented [7]. However, nowadays, HPLC‐MS/MS or UHPLC‐MS/MS, are described as the most sensitive, selective, rapid, and robust analytical methods for the analysis of these glycopeptides [1–2, 8–9]. It is significant to be mentioned that, for the determination of TEIC and VANC in serum, the fluorescence polarization immunoassay has been considered the most common analytical method. Nonetheless, compared to the HPLC method, it is a less reproducible, complex, and non‐cost‐effective method [10].

An important step prior to the analysis of drugs in biological fluids using sensitive analytical methods such as HPLC‐MS is the proper sample treatment. The preferred treatment method is SPE for removing endogenous interferences from difficult matrices and preconcentrating the target analytes. Based on SPE theory, the extracted components are distributed between a liquid mobile phase (organic solvents or water) and a solid stationary phase, depending on their physicochemical properties [11]. According to the literature, various types of stationary phases such as silica or polymeric sorbents were used. In addition to that, for the treatment of VANC and TEIC in biological samples, other treatment techniques such as protein precipitation (PP), liquid‐liquid extraction, and ultrafiltration were used [11, 12].

From 2007 up to 2017, most of the studies dealt with the development of analytical methods for the single determination of VANC [1, 4, 7, 11]. In contrast, fewer studies have dealt with the analysis of TEIC [2, 10, 13] or the simultaneous determination of the two glycopeptides in biological samples [11, 14].

Taking into consideration that most of the existing analytical methods focus on a single analysis of VANC or TEIC, the main objective of this research work was to develop a sensitive analytical method using HPLC coupled with both photodiode array and mass spectrometry (HPLC‐PDA/MS) for the simultaneous determination of VANC and TEIC in human urine. Additionally, for the treatment of the urine samples, an SPE protocol was developed and optimized, making the specific analytical method more sensitive and reliable for the simultaneous determination of these glycopeptides in difficult matrices such as urine samples.

It is worth mentioning that this research work is the first attempt to use an HPLC‐PDA system coupled with an MS method for the simultaneous determination of VANC and TEIC in urine samples using data from both detection methods. In the present research work, a comparison of the two detection methods was performed and the comparative data of the validation of the analytical methods were presented.

Moreover, the importance of this research effort is because the collection of urine might be used as a non‐invasive alternative sampling method in contrast with blood and serum sampling. It is an important advantage of the analytical protocol, especially for patients with several diseases, when blood sampling is difficult. As is shown from the literature review, most of the research works described the pharmacokinetic study of the specific antibiotics in plasma [2, 15].

## 2 MATERIALS AND METHODS

### 2.1 Chemicals and reagents

Vancomycin hydrochloride and TEIC were purchased from Sigma‐Aldrich (St. Louis, MO, USA) while methanol (MeOH), water (H_2_O), ACN (CH_3_CN), and TFA were HPLC gradient LiChrosolv (Merck Millipore, Massachusetts, USA). EDTA disodium salt (Na_2_EDTA), formic acid (FA), and ammonium hydroxide (NH_4_OH) were analytical grade and purchased from Sigma‐Aldrich (St. Louis, MO, USA).

### 2.2 Equipment

A system of HPLC‐PDA/MS consisting of Waters Alliance HPLC system (e2695) with a PDA detector (PDA 2998), equipped with Waters ACQUITY QDa mass spectrometer was used for chromatographic analysis of the samples. For the Mass Spectrometry analysis, a positive ion mode ESI was carried out.

For the chromatographic separation, a C18 column (4.6 × 150 mm Symmetry C18, 5 μm) was selected with a temperature adjusted to 40°C. The flow rate was 0.8 mL/min, and the injection volume was 10 μL. The used mobile phases were 0.1% TFA in water (eluent A) and 0.1% TFA in MeOH (eluent B), followed by gradient elution. Initial conditions of 95% Α%–5% Β was retained for 2 min, followed by a change to 50% Α%–50% Β within 6 min. Subsequently, the initial ratio of 95% Α–5% Β was reappearance within 7 min, and this composition was kept for 3 min. The run time of analysis was 10 min. For PDA, the UV detection wavelength was 240 nm. For MS detection capillary voltage settings were: Pos: 1.0 kV, Neg: 0.8 kV, gain 1, probe 600°C. The optimum ionic mass fragments were *m/z* 725.75 *m/z*for VANC and *m/z* 782.65 *m/z*for TEIC with cone voltages of 15 and 12 V, respectively.

### 2.3 Sample preparation

Stock solutions of VANC and TEIC were prepared in a mixture of water and MeOH, 70:30, v/v. The working solutions at a concentration of 1000 mg/L of the individual compound were prepared by appropriate dilutions of the stock solutions in a mixture of water and MeOH 70:30 v/v.

The stock solutions were stored at −20°C, while the working solutions were refrigerated to 5°C. Calibration standard solutions were prepared from the working solutions at concentrations 0.25, 0.5, 1, 3, 5, 10, 20, 30, 40, and 80 mg/L in water and MeOH in ratio 75:25, v/v. Quality controls solutions were prepared at 40, 15, and 1 mg/L for VANC and 80, 40, and 2 mg/L for TEIC in a mixture of water/MeOH in a ratio of 75:25, v/v.

### 2.4 Urine sample preparation

The urine was selected as a working matrix for the following reasons: (1) the collection of urine is a non‐invasive sampling method and (2) the glycopeptides can be excreted abundant in the urine, resulting in a high concentration of them in it, in contrast to the other biological fluids [16].

Urine samples were collected from a healthy individual who had not been treated with VANC or TEIC for more than 1 year. The samples of urine were collected in sterile containers and immediately were frozen at –20°C to avoid a possible alteration of the sample during transport and storage of the samples. It is significant to be mentioned that the urine samples were analyzed within 24 h after they were collected. For the extraction of the specific glycopeptides, urine samples were spiked with low and high concentrations of the two antibiotics.

The used urine samples were a mixture of 3 mL of blank urine with 1 mL of ACN and 5% trichloroacetic acid (TCA) which were spiked with standard solutions of two glycopeptides resulting in samples with high and low concentrations of VANC (1 and 40 mg/L) and TEIC (2 and 80 mg/L). For the precipitation of proteins, the prepared mixtures were added in 15 mL reaction tubes and were centrifuged at 4000 rpm for 3 min. Centrifugations were performed in a high‐volume centrifuge from centurion Scientific (West Sussex, UK) (Rotor BRK 5510). After the centrifugation of the urine mixture, the supernatant directly was loaded to the cartridges of SPE.

### 2.5 Solid‐phase extraction

For the development of the optimized SPE protocol, blank urine samples spiked with VANC and TEIC in low (1 and 2 mg/L, respectively) and high concentrations (40 and 80 mg/L, respectively) were used.

Sample extraction was carried out using Oasis HLB (3cc, 60 mg sorbent, 60 μm particle size) cartridges (Waters, Milford, MA, USA). Conditioning of cartridges was performed consecutively with 5 mL CH_3_OH and 5 mL H_2_O. Both the working solutions of 5 mL and urine samples were loaded to cartridges by gravity, washed with 1 mL H_2_O, and vacuum dried with air for 10 min. Analytes were eluted using 6 mL of CH_3_OH. The extracts were evaporated to dryness at 35°C under a gentle stream of nitrogen and reconstituted with 25% CH_3_OH and 75% H_2_O in the final volume of 1 mL vial. The reconstituted solution of 10 μL was injected into the HPLC‐PDA/MS system for analysis.

### 2.6 Validation of the analytical method

The assay of VANC and TEIC in urine samples was validated according to the published European Medicines Agency (EMA) pertinent recommendations [17].

#### 2.6.1 Linearity

Method linearity was assessed by calculating the coefficient of determination (*R* ^2^), y‐intercept, and slope of the regression line obtained from matrix calibration curves. For the establishment of linearity, seven calibration standard solutions were prepared by spiking the blank samples of urine with VANC and TEIC, giving solutions with final concentrations ranging from 0.25 to 80 mg/L, after the use of the SPE method [17].

#### 2.6.2 Accuracy and precision

For the validation of the assay, the precision was studied in two means: repeatability and intermediate precision. The precision of the analytical procedure was considered using the coefficient of variation (%CV) of a series of measurements with an acceptable range below 15% [14]. Intra‐day and intermediate precision were studied by analyzing standard solutions, named Quality control samples at three different concentrations of 1, 15, and 40 mg/L for VANC and 2, 40, and 80 mg/L for TEIC. For the calculation of intra‐day precision and inter‐day precision, three quality control levels were analyzed five times on the same day and five times on five different days, respectively.

The accuracy of the analytical methodology was performed by studying the total extraction recovery for each analyte in urine samples. The % recovery of each analyte was estimated at a low (1 mg/L VANC and 2 mg/L TEIC)) and a high concentration (40 mg/L VANC and 80 mg/L TEIC) (*n* = 5 for each level) of them. Analyte‐free human urine samples were spiked with analytes at different concentrations, prior to the SPE method. Total extraction recovery (%) was calculated by comparing the average concentration of the extracted samples with unextracted standards of VANC and TEIC.

#### 2.6.3 LOD and LOQ

For the definition of LOD and LOQ, different approaches can be used, such as a non‐instrumental or instrumental approach. According to EMA, the LOD is the lowest concentration of the analyte in the sample that can be detected, and LOQ is the lowest concentration of the analyte that can be quantified with precision and accuracy according to the test condition [17].

The LOQs for both analytes were estimated based on the response of five replicate calibrator standards at the lowest concentration in urine of the calibration range (0.25 mg/L for VANC and 1 mg/L for TEIC) with ≤20% precision [13]. The LOD was estimated by analyzing blank samples spiked the analytes with concentrations lower than 0.25 mg/L and 1 mg/L for VANC and TEIC, respectively, and was defined as the lowest concentration of analyte for which S/N was 3 [14].

#### 2.6.4 Stability

For the evaluation of the stability of VANC and TEIC in urine, long‐term stability tests (for 15 days) at –20°C and short‐term stability tests (for five consecutive days) at 4°C, were performed. For the study of the long‐term stability, two QC samples in different concentrations were prepared and stored at –20°C for 15 days, while for the short‐term stability, two QC samples with the same concentrations were prepared and kept at 4°C. It is noteworthy to be mentioned, that during the short‐term study, the target analytes were determined at room temperature for five consecutive days. The acceptance criteria should be ≤15% deviation.

## 3 RESULTS

### 3.1 Optimization of chromatographic and mass spectroscopic conditions

For the development of the analytical protocol, various chromatographic and mass spectroscopic parameters such as the type of the column, the column temperature, the mobile phase, the flow rate, the elution system, as well as the desired ion fragments with the appropriate cone voltage were studied [14, 18–20].

According to the literature review, the simultaneous determination of TEIC and VANC in biological fluids using Liquid Chromatography coupled with the UV method is not so common [21]. In contrast, in recent years, the most used detection method for the determination of both glycopeptides is MS [21]. As already mentioned in this research work, for the simultaneous determination of VANC and TEIC in urine, an analytical method based on the HPLC tandem PDA and MS method was developed. It is worth mentioning that the usage of HPLC‐MS with a PDA detector was only used for the determination of VANC in serum [20]. As Tsai et al. reported, the simultaneous analysis of the two glycopeptides was performed using a UHPLC‐MS/MS system with gradient elution using FA in water and MeOH or MeOH and FA aqueous solution [22].

#### 3.1.1 Chromatographic separation

For the development and optimization of the analytical protocol using the HPLC‐PDA/MS system, various type of columns was examined [4, 12]. Among the tested analytical columns (Symmetry, Hypersil BDS and Hypersil GOLD), the Symmetry, C18 analytical column showed the greatest performance for the analysis of the two glycopeptides. The optimized column temperature was set at 40°C [23].

For the selection of the optimized chromatographic conditions, both isocratic and gradient elution systems were studied. The separation of the drugs was obtained by gradient elution as described above. Various solvents were examined such as water, MeOH, ACN, mixtures of MeOH/ACN/acetone, and ACN/2‐propanol. Moreover, acidic conditions were studied with the addition of a small proportion of acid (FA, TFA, or some salt with predominant ammonium acetate or phosphate salt) in the eluents.

A system of mobile phases, 0.1% TFA in water and 0.1% TFA in MeOH was selected. It is noteworthy that this combination of the mobile phases was not reported in previous research works [11, 12]. The flow rate of the mobile phases was set at 0.8 mL/min and the run time was equal to 10 min (Figure S1).

#### 3.1.2 MS analysis

The mass parameters such as cone voltage and *m/z* values in positive ion ESI mode through the direct infusion of a standard solution of VANC and TEIC in a mixture of MeOH: water (75:25, v/v) were investigated. Initially, a full scan mode with a scan range of *m/z* 50–1249.00 and cone voltage set at 15 V, was performed. The cone voltages were studied for collecting the most abundant product ions. For the quantification of the glycopeptides, the most sensitive fragment was selected.

**FIGURE 2:**
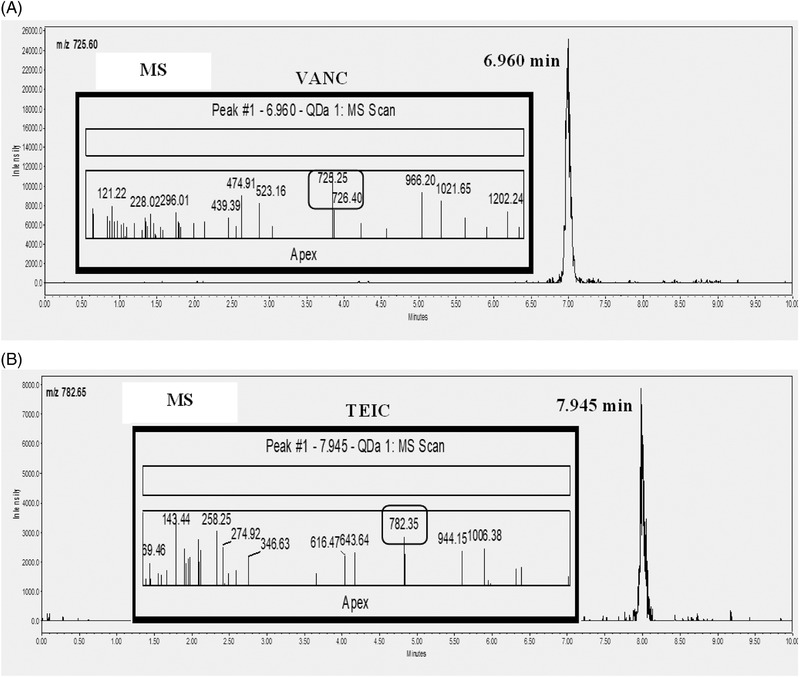
Chromatograms of a mixture of vancomycin and teicoplanin at a concentration of 10 mg/L using MS data (A) vancomycin (VANC) fragment ion at MS (*m/z* 725.25) and (B) teicoplanin (TEIC) fragment ion at MS (*m/z* 782.35)

As it is known, the VANC and TEIC in the positive ESI mode can be easily protonated to form doubly charged protonated molecular ions due to the structural‐functional groups which include nitrogen (Figure 2A,B, internal diagrams) [14, 12, 23,]. The monitored ions were *m/z* 725.35 and 782.35 for VANC and TEIC, respectively. These compounds present multiple basic ionizable groups and the deprotonated molecule, [M+2H]^2+^ provided the most sensitive signal. It is noteworthy to be mentioned that the quantification of VANC and TEIC was performed using data from both the selected ion recording (SIR) and the absorbance at 240 nm. For the selection of the optimum fragment ion, the proposed fragmentation pathway of each compound was studied. Cone voltage values were optimized for each of the two selected fragment ions, at 15 V for VANC and 12 V for TEIC. Capillary was set as follows: Pos: 1.0 kV, Neg: 0.8 kV, gain 1, probe 600°C.

The extracted ion chromatogram and the MS fragmentation patterns of VANC and TEIC are shown in Figure 2. These diagrams showed that VANC and TEIC eluted at 6.966 and 7.976 min with 725.25 *m/z* and 782.35 *m/z* as doubly protonated molecular ions [M + 2H]^2+^ of the precursor ion of VANC and TEIC A3‐1, respectively.

**FIGURE 3:**
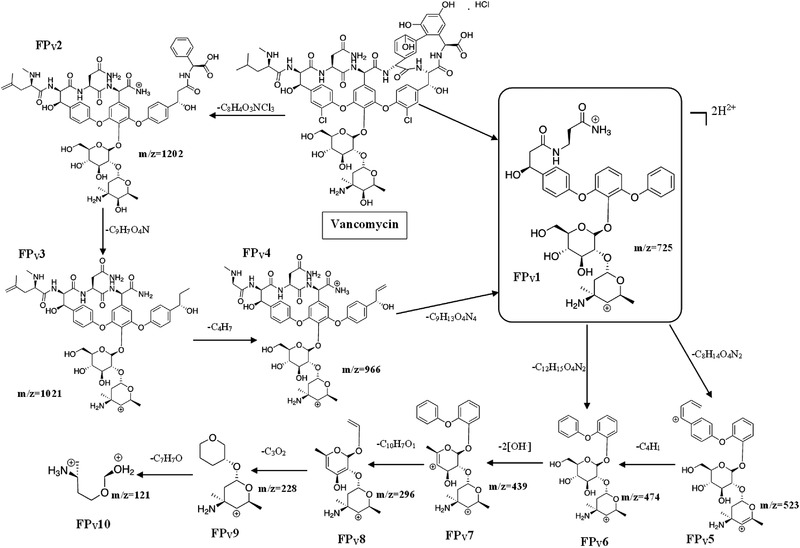
The proposed elucidation pathway of vancomycin

Figure 3 illustrates the proposed fragmentation pathway of VANC with the main fragment ion at *m/z* 725.25. As it is known, both VANC and TEIC present complex chemical structures. The structure of VANC is a combination of a polypeptide and an aminoglycoside.

According to the proposed fragmentation pathway of VANC, it was initially fragmented into the two main fragment ions, *m/z* 725.5 (***FP_V_1***) which was used for the quantification, and *m/z* 1202 (***FP_V_2***). A series of characteristic fragmentation ions created at *m/z* 1021 [FPv2‐(C_9_H_7_O_4_N)]^+^ (***FP_V_3***), 966 [FPv3‐(C_4_H_7_) or FPv1+(C_9_H_13_O_4_N_4_)]^+^ (***FP_V_4***), 523 [FPv1‐C_8_H_14_O_4_N_2_)]^+^ (***FP_V_5***), 474 [FPv1‐(C_12_H_15_O_4_N_2_)]^+^ (***FP_V_6***), 439 [FPv6‐2(OH^–^)]^+^ (***FP7***), 296 [(FPv7‐(C_10_H_7_O_1_)]+ (***FP_V_8***), 228 [FPv8‐(C_3_O_2_)]^+^ (***FP_V_9***), and 121 [FPv9‐(C_7_H_7_O)]^+^ (***FP_V_10***). According to the proposed fragmentation pattern, the fragmentation occurred mainly with the decomposition of the polypeptide, in contrast to the part of the aminoglycoside which was steady. Only the fragmentation product of *m/z* 121 was a result of the decomposition of the aminoglycoside of VANC.

The structure of all the types of TEIC presents the same glycopeptide core, which is a combination of two carbohydrates: mannose and *N*‐acetylglucosamine. Τhe structure of the TEIC A3‐1 comprises a macropolycyclic heptapeptide including a phenolic hydroxy group which has been converted to alpha‐D‐mannoside. The secondary alcohol group has been converted to the corresponding 2‐acetamido‐2‐deoxy‐beta‐D‐glucoside.

**FIGURE 4:**
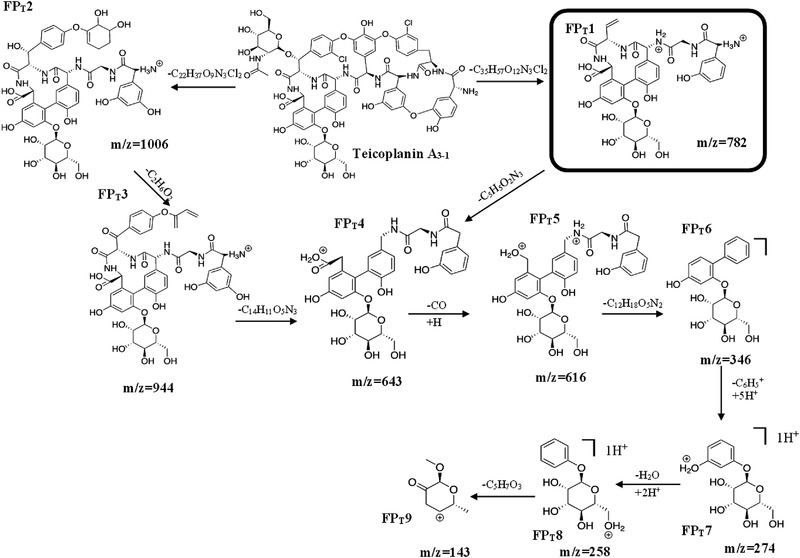
The proposed elucidation pathway of Teicoplanin A_3‐1_

The elucidation of the fragmentation pathway of TEIC is presented in Figure 4. As is shown in the proposed fragmentation pattern of TEIC, all the fragments present the a‐D‐mannose part which is very steady in contrast with the part of N‐acetylglucosamine.

Like the proposed fragmentation pathway of VANC, a series of characteristic fragmentation ions of TEIC created at *m/z* 1006 [**A_3‐1_**‐(C_22_H_37_O_9_N_3_Cl_2_)]^+^ (***FP_T_2***), 944 [***FP_T_2***‐(C_2_H_6_O_2_)]^+^ (***FP_T_3***), 643 [***FP_T_3***‐C_14_H_11_O_5_N_3_)]^+^ or [***FP_T_1***‐C_5_H_5_O_2_N_3_]^+^ (***FP_T_4***), 616 [**FP_T_4**‐(CO)+H^+^]^+^ (***FP_T_5***), 346 [***FP_T_5***‐C_12_H_18_O_5_N_2_]^+^ (***FP_T_6***), 274 [(***FP_T_6***‐(C_6_H_5_^+^)+5H^+^]^+^ (***FP_T_7***), 258 [***FP_T_7***‐(H_2_O)+2H^+^]^+^ (***FP_T_8***), and 143 [***FP_T_8***‐(C_5_H_7_O_3_)]^+^ (***FP_T_9***).

### 3.2 Optimization of SPE procedure

The direct injection of the samples into the HPLC column is considered a cost‐effective method not only for reducing the sample pretreatment time but also for diminishing the errors which are associated with sample handling [13]. However, clogging of the HPLC column due to the abundance of proteins in urine such as albumin is one of the main disadvantages of direct injection.

For this reason, an analytical protocol based on SPE was developed. As it is known, one of the most critical steps in the determination of multi‐target analysis of drugs with similar physicochemical properties is the extraction procedure from difficult matrices such as biological fluids. For the determination of organic compounds in various biological fluids like urine, the SPE is the preferable pretreatment method due to the matrix effect. Despite its advantages, the SPE procedure also shows disadvantages like the various experimental steps, making it, a time‐consuming method, especially when many urine samples are analyzed. Nevertheless, the SPE procedure is one of the effective pretreatment methods of biological fluids especially urine, not only for removing the endogenous proteins but also for the preconcentration of the target analytes.

One of the main objectives of the specific research work was to develop a simple pretreatment process for the simultaneous separation of the two glycopeptides in urine, showing good repeatability and accuracy. According to the literature review, the precipitation of protein, followed by the SPE procedure was the main pretreatment process of the biological fluids [1, 11–12]. In this research work, besides the PP through centrifugation, the SPE procedure was followed. For the development of the optimized SPE, various experimental conditions, such as conditioning solvents, elution solvents, and pH of the solvents were studied.

According to Cass et al., a different method based on the online extraction of VANC in urine was used. However, this method was considered a cost‐ineffective method due to the combination of three columns: one extraction column and two other analytical columns (for separation and equilibration) [24]. In contrast, Javoska et al. proposed a different pretreatment procedure based on simple PP and dilution [1]. One of the main problems using the ESI ionization technique is the signal enhancement and suppression of the target analytes due to the complexity of the matrix. The biological fluids such as urine are very complex matrices due to the presence of a huge number of compounds such as proteins and cells, that can affect the identification and quantification of the target analytes.

It is significant to be mentioned, that prior to the application of SPE, direct injection of the diluted sample into the chromatographic column was performed. Dilutions 1:2, 1:5, 1:10, and 1:20 (v sample/v mobile phase) were tested. As it was reported, the dilution of the sample improves the effect of the matrix but also reduces the sensitivity of the method resulting in low recoveries of both glycopeptides. Unfortunately, despite the dilution of the urine samples, a matrix effect for VANC was observed. In contrast, TEIC did not observe the same phenomenon. For VANC, the slope ratio was presented as lower than 0.8 (slope < 0.8), showing a strong matrix suppression effect.

**FIGURE 5:**
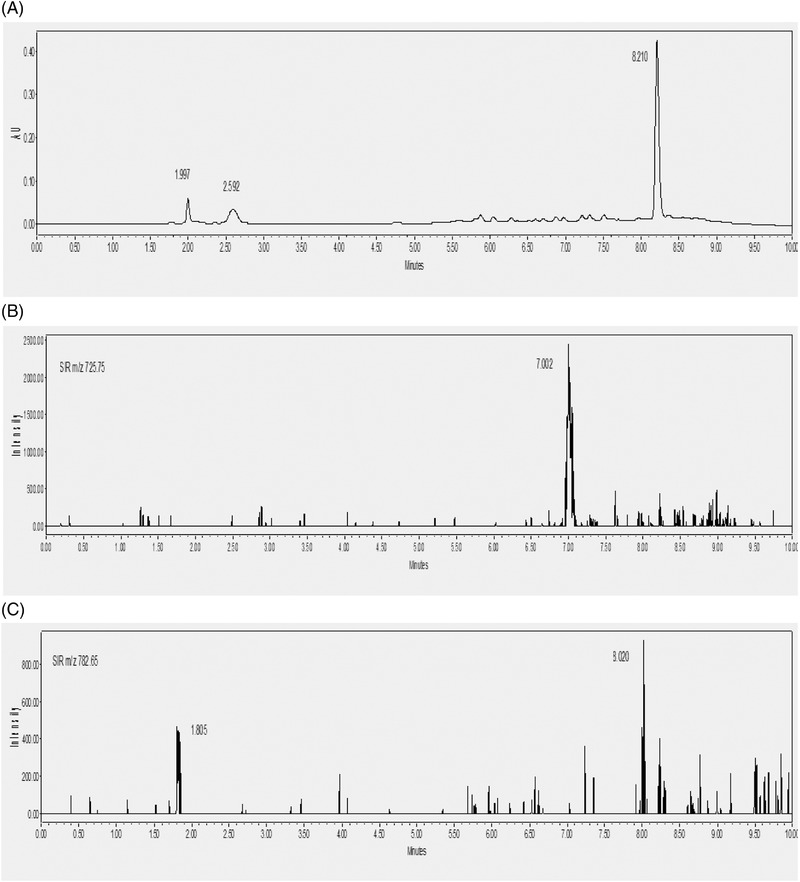
(A) Chromatograms of the mixture of vancomycin and teicoplanin at a concentration of 10 mg/L in urine (B) vancomycin fragment ion at MS (*m/z* 725.25), and (C) teicoplanin fragment ion at MS (*m/z* 782.35)

The obtained PDA chromatograms showed a sharp peak at 8 min which was not corresponded either to VANC or TEIC (Figure 5). The corresponding MS spectrum illustrated different fragment ions at *m/z* 693.21, 932.75, and 1160.99. In contrast, the SIR chromatograms showed the presence of the VANC and TEIC in the urine sample, giving the corresponding fragment ions at *m/z* 725 and 782, respectively. The chromatographic peak at 8 min was probably due to the presence of the endogenous proteins. For the optimization of the extraction protocol, the precipitation of proteins in urine was preceded. According to the literature review, the PP is the most widely used method for the determination of VANC and TEIC in various biological fluids [1, 11–12, 14, 16]. The precipitation of proteins was selected because is not only a simple method but is also a time‐saving and cost‐effective method. Furthermore, the usage of organic solvents or acids for the denaturation of proteins of biological fluids prevents interference in the analysis, resulting in a longer lifetime of the column [12]. For this reason, we investigated the use of various polar and nonpolar solvents, for the separation of VANC and TEIC from the urine proteins. Using solvents with different polarities, the hydrophobic components such as TEIC were separated from the urine proteins. In contrast, the hydrophilic components such as VANC are too polar to be separated from the proteins with large molecular weights such as albumin [12, 18].

The precipitation of proteins from urine samples with the addition of an appropriate organic solvent such as ACN or acid followed by centrifugation is a simple and efficient method [12]. For the reduction of proteins, according to Cheng and co‐workers, a combination of acetone and TCA in urine was added [16]. In our research work, the addition of the mixture of acetone and TCA in urine did not reduce the proteins, nor the chromatographic peak at 8 min. The ACN and TCA are frequently used as precipitation agents of proteins in biological fluids such as plasma, serum, and urine [1, 6, 9–10, 24]. However, the single addition of ACN or TCA into the urine samples did not achieve a good recovery of VANC and TEIC, showing a strong matrix effect [15]. For this reason, we studied the synergistic effect of the solvents on the precipitation of proteins. In the usage of organic solvents, the recoveries of the glycopeptides were less than 30% due to the low solubility of the peptides in organic solvents. For this reason, the addition of chlorine‐containing acid for inducing the precipitation of proteins has been used. The TCA was selected because according to the bibliography the precipitation of protein is not affected by pH but by the trichloro group [16]. The use of the combination of ACN and TCA resulted in better recoveries for the peptide compounds. A combination of ACN and 5% TCA was tested [25]. This mixture of solvents was selected as a precipitation agent of proteins in real samples because has exhibited a better extraction purity than the other mixtures. More specifically, for the precipitation of proteins, a mixture of 3 mL of a blank urine sample with 1 ml of ACN and 5% TCA was spiked with the proper concentration of VANC and TEIC and then was centrifuged at 4000 rpm for 3 min.

**FIGURE 6:**
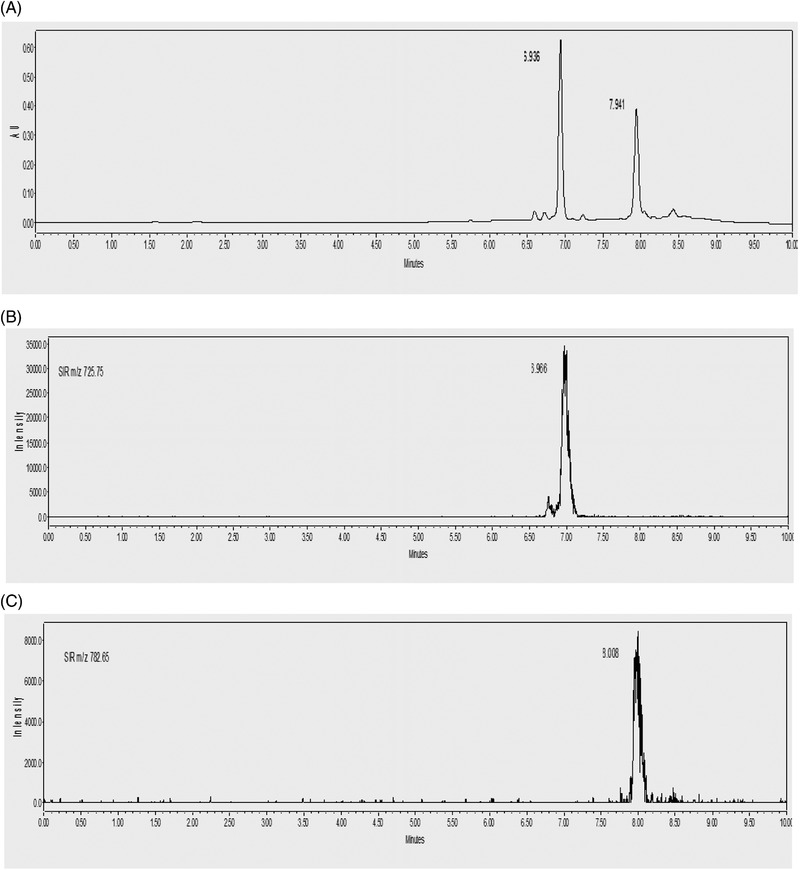
(A) Chromatograms of the mixture of vancomycin and teicoplanin at a concentration of 10 mg/L in urine after protein precipitation and SPE, (B) vancomycin fragment ion at MS (*m/z* 725.25), and (C) teicoplanin fragment ion at MS (*m/z* 782.35)

For the optimization of the SPE method for the simultaneous extraction of VANC and TEIC in urine, several different experimental parameters such as the type of cartridges were studied. According to the literature, for the pretreatment of the biological fluids using SPE, several different types of sorbents such as polymeric sorbents with lipophilic‐hydrophilic properties were used [14]. As it is known, the extraction procedure is an important step due to the complexity of the matrix of the biological fluids and the different physicochemical properties of the drugs. For this reason, the development and optimization of extraction protocols for the simultaneous extraction of the glycopeptides should be performed for covering a wide range of polarity in urine to reduce the sample handling [14]. The OASIS HLB cartridge was applied more than the other sorbents due to the combination of a hydrophilic *N*‐vinyl‐pyrrolidone and a lipophilic divinylbenzene. Due to this peculiarity of this adsorbent, these cartridges are suitable for the extraction of both glycopeptides, VANC and TEIC from urine because their structures have lipophilic and hydrophilic moieties. In addition to that, the OASIS HLB cartridges present significant stability in a wide range of pH values, showing an ideal behavior for the extraction of acidic, basic, and neutral target analytes [26]. In this research work, only the cartridges of Oasis HLB were evaluated. The cartridges were conditioned first with 5 ml of MeOH and then with 5 ml of water. After that, the step of loading the samples of urine through the cartridges was followed. Several solvents such as water, acidic water (with 0.1% FA), basic water (with 0.1% NH_4_OH), and Na_2_EDTA were used as washing solvents, during the rinsing step. For the rinsing step, water was selected as an optimum washing solvent. Finally, after drying the cartridges for 20 min, was followed by elution. Different solvents such as a MeOH, a 1:1, v/v mixture of MeOH: water, and acidic MeOH (with 0.1% FA) were examined. For the optimized SPE protocol, the elution step was performed using 6 mL MeOH (Figure 6).

### 3.3 Validation

For the validation of the proposed analytical methodology, the linearity, intra‐day and inter‐day precision, and the accuracy, as well as the LODs and LOQs were studied. It has to be mentioned that for the validation of the method, both data from PDA and MS detection were used.

#### 3.3.1 Linearity

For the study of the linearity, the mean calibration curves (*n* = 3) of three different measurements for VANC and TEIC in urine were constructed.

The R^2^ value was equal to 0.9990 and 0.9995 from PDA data and 0.9981 and 0.9949 from MS data, for VANC and TEIC, respectively. Τhese values are within the acceptable range of 0.98 to 1, showing good linearity of the method for both glycopeptides in urine, since the coefficient of determination (*R* ^2^) tends to value 1 [17].

#### 3.3.2 Precision

To evaluate intra‐day precision, three concentration levels of spiked blank urine samples of VANC and TEIC were analyzed five consecutive times (*n* = 5) over a day. The %CV for VANC ranged from 0.16 to 4.90 and 2.31 to 6.56 while for TEIC from 0.33 to 4.03 and from 0.19 to 3.59, for PDA and MS data, respectively (Table 1).

**TABLE 1:** Results of the study of intra‐day and inter‐day precision and accuracy of vancomycin and teicoplanin using both detection methods, PDA and MS

	**Nominal concentration (mg/L)**					
	**Vancomycin**			**Teicoplanin**		
**Compound**	**1**	**15**	**40**	**2**	**40**	**80**
***With PDA***	**Intra‐day, *n* = 5**					
Mean concentration ± SD	1.25 ± 0.02	15.59 ± 0.76	42.28 ± 0.07	2.46 ± 0.11	40.16 ± 0.23	79.78 ± 0.94
%CV	1.64	4.90	0.16	0.33	1.03	4.03
***With MS***						
Mean concentration ± SD	1.17 ± 0.03	15.30 ± 1.00	43.01 ± 0.99	2.68 ± 0.1	41.87 ± 0.08	80.99 ± 1.97
%CV	2.75	6.56	2.31	3.59	0.19	2.43
***With PDA***	**Inter‐day, *n* = 5**					
Mean concentration ± SD	1.20 ± 0.07	14.76 ± 0.40	41.52 ± 1.27	2.48 ± 0.15	40.01 ± 0.17	81.05 ± 1.72
%CV	6.05	2.70	3.06	6.00	0.43	2.12
***With MS***						
Mean concentration ± SD	1.56 ± 0.06	15.96 ± 1.12	43.00 ± 1.01	2.93 ± 0.20	41.00 ± 0.38	82.57 ± 1.88
%CV	4.07	7.03	2.34	6.70	0.94	2.27
	**Accuracy**					
***With PDA***						
Recovery (%)	64	–	91	104	–	121
***With MS***						
Recovery (%)	83	–	97	92	–	113

The inter‐day precision was evaluated by the analysis of the samples of VANC and TEIC at the same concentration levels as for the study of the intra‐day precision on five consecutive days (*n* = 5). It is significant to be mentioned, that for the inter‐day precision study, the samples were prepared daily. As shown in Table 1, the % CV for the study of inter‐day precision for VANC was estimated from 2.70 to 6.05 and from 2.34 to 7.03 and for TEIC from 0.43 to 6.00 and from 0.94 to 6.70, for PDA and MS methods, respectively.

The intra‐day and inter‐day precisions were acceptable according to the criteria of the precision since the %CV values were lower than the acceptable threshold of 10% in all cases. As is shown in Table 1, the higher rate was 7.03% showing good repeatability.

#### 3.3.3 Accuracy

The accuracy of the developed analytical method was studied by the estimated extraction recoveries using the peak area of VANC and TEIC in spiked blank urine and standard solutions at two levels of concentration, 1 and 40 and 2 and 80 mg/L, respectively.

Obtained average extraction recoveries for VANC, ranged from 64 to 91% and from 83 to 97%, while for TEIC from 104 to 121% and from 92 to 113%, using the PDA and MS methods, respectively (Table 1). These values of extraction recovery indicated a good accuracy. However, some of the recovery values using the PDA detection method were out of the range of the acceptable limits of 80–120%. In contrast, using the MS detection method, the recovery of these glycopeptides was within acceptable limits.

#### 3.3.4 Stability

As presented in Table 2, both VANC and TEIC were stable at –20°C for 15 days and after freezing and thawing for five consecutive days. During the stability study, for both VANC and TEIC, the % CV was lower than 11%, showing a non‐systematic loss in sensitivity. Stock solutions proved to be stable in freeze storage conditions at ‐20°C as the estimated values of % CV of VANC and TEIC were found to range between 1.10 and 10.21 and between 5.00 and 7.48, respectively.

**TABLE 2:** Stability data for vancomycin and teicoplanin in urine samples (two QC levels)

**Nominal concentration (mg/L)**	**Stability test**	**Mean concentration ± SD**	**%CV**	**Accuracy (%)**
Vancomycin				
1	Five consecutive days	1.15 ± 0.084	7.27	115
	15 days (−20°C)	0.91 ± 0.09	10.21	91
20	Five consecutive days	19.97 ± 0.22	1.10	99.5
	15 days (−20°C)	18.72 ± 1.66	8.89	94
Teicoplanin				
2	Five consecutive days	1.82 ± 0.10	5.49	91
	15 days (−20°C)	1.78 ± 0.13	7.48	89
40	Five consecutive days	39.95 ± 2.00	5.00	99
	15 days (−20°C)	38.23 ± 1.93	5.05	96

Moreover, the study of the long‐term stability showed accuracies between 91–94 and 89–96%, for both VANC and TEIC in urine, respectively. In the short‐term stability study, the two glycopeptides also achieved the same level of accuracy, 99.5–115% for VANC and 91–99% for TEIC. These results demonstrated that the specific analytical method was still sensitive under these conditions since VANC and TEIC were stable molecules.

#### 3.3.5 Determination of LOD and LOQ

The LOD and LOQ were calculated by estimating the standard deviation of five different spiked blank urine samples with VANC at a concentration of 0.25 mg/L and TEIC at a concentration of 1 mg/L, respectively. Table 3 illustrates the LOD and LOQ values of the two glycopeptides which were estimated using both the PDA and MS methods.

**TABLE 3:** Validation parameters (Linearity, LOD, and LOQ) of the optimized HPLC–PDA/MS method in urine

**Compound**	**Linearity range (mg/L)**			
Vancomycin	0.25 ‐ 40		0.3–40	
	***PDA***		***MS***	
	*LOD (mg/L)*	*LOQ (mg/L)*	*LOD (mg/L)*	*LOQ (mg/L)*
	0.076	0.25	0.1	0.33
	**Linearity range (mg/L)**			
Teicoplanin	1–80		2–80	
	***PDA ***		***MS***	
	*LOD (mg/L)*	*LOQ (mg/L)*	*LOD (mg/L)*	*LOQ (mg/L)*
	0.33	1.1	0.64	2.1

The LOD and LOQ values for VANC were 0.076 and 0.25 mg/L using the PDA method and 0.1 and 0.33 mg/L, using the MS method, while for TEIC were 0.33 and 1.1 mg/L and 0.64 and 2.1 mg/L, respectively. As it is shown, the LOD and LOQ values of VANC were 4–6 times lower than the values of TEIC, for both detection methods. These data indicated that the developed method shows lower LOQ for VANC rather than TEIC. Nevertheless, the developed analytical method shows low values of LOQ for both glycopeptides in urine. Table 4 gives examples of an overview of the LOD and LOQ values of glycopeptides in urine from the bibliography. As is shown in Table 4, these LOD and LOQ values are in the same order of magnitude or lower compared to the bibliography, showing that the developed method can be used for analyzing glycopeptides in low concentrations [14]. Moreover, as the data shows, the PDA method has indicated lower values of LOD and LOQ than the MS method.

**TABLE 4:** Examples of an overview of LOD and LOQ of glycopeptides in urine

**Matrix**	**Analytical Method**	**Preparation method**	**LOD (mg/L)**	**LOQ (mg/L)**	**Ref**.
Urine	UHPLC‐MS/MS	Dilution	0.9	2.97	1
Urine	HPLC‐UV_215 nm_	SPE	0.11	0.36	12
Urine	UHPLC‐MS/MS	Dilution	0.3	1	14

## 4 DISCUSSION

In this research work a novel analytical method based on the HPLC using both PDA and MS methods for the simultaneous determination of two antimicrobial agents, VANC and TEIC in urine, was developed. The specific analytical method was validated using urine samples in terms of selectivity, sensitivity, accuracy, precision, and stability. The validation parameters were within the set specifications according to EMA, showing the accuracy and selectivity of the specific method.

According to Begg et al., the determination of the concentration of glycopeptides such as VANC in the body is necessary, due to the need for the estimation of the adequate dosing of antibiotics [27]. For this reason, until recently the serum has been used as a matrix for the estimation of its concentration. Due to the association of the cases of poor clinical outcomes with the low levels of VANC in serum, in this research work, we tried to develop an analytical method using urine as a substitute for serum, taking into consideration the benefits of using urine, such as the less costly sampling and the easier way to manage the urine in contrast with serum or blood samples.

Although the developed method was not applied for the determination of VANC and TEIC in biological samples of patients, the low values of LOD and LOQ of this method show its suitability to be applied for the pharmacokinetic study in patients. Α trough concentration (C_min_) of glycopeptides for treating the majority of severe infections is > 10 mg/L. The study of the minimum inhibitory concentration in VANC and TEIC, has forced guidelines that advocate a target trough concentration (C_min_) of 15–20 and >15 mg/L for VANC and TEIC, respectively, especially in patients with methicillin‐resistant *S. aureus* infections [28]. Considering the trough concentrations of TEIC and VANC and the concentration of drugs in urine that have been reported, the HPLC‐PDA/MS method is adequate for the quantification of the specific drugs for the ongoing population pharmacokinetic study [28].

Moreover, the developed method satisfies the necessity for the simultaneous determination of antimicrobial agents in biological fluids such as urine due to the simplicity, sensitivity, and accuracy of the method. This is justified based on the previous studies where the VANC and TEIC in various biological fluids were determined at concentrations ranging from 7 to 35 mg/L [8, 14–15, 19] which are higher than the LOD and LOQ of the developed method. According to Cazorla‐Reyes et al., the LOD and LOQ of VANC in urine were 0.3 and 1 mg/L, respectively [14]. This proves the sensitivity of the present analytical method since the LOD and LOQ values were six times lower than the reported values.

As previously reported in the literature, the LC‐MS/MS has been recognized as a powerful technique for the accurate identification and quantification of several drugs and metabolites in various biological fluids [23]. Most of the analytical methods have cited the development of chromatographic methods such as HPLC‐UV and HPLC‐MS/MS, accompanied by protein precipitation in urine followed by time‐consuming SPE [1, 6, 8, 23].

One important aspect of this research work is the simultaneous determination of the two antimicrobial agents with many similarities in their structure using HPLC with PDA and MS in series. The two detection methods have shown almost the same accuracy and sensitivity. Also, the data from the simultaneous use of both detection methods enhanced the reliability of our developed analytical method for the determination of the two antimicrobial compounds. Our data for the determination of the two glycopeptides by two different modes, PDA and MS (full scan and SIR mode) analysis, showed the same order of selectivity and sensitivity as a multiple reaction mode (MRM) on triple quadrupole mass spectrometry (LC‐MS/MS), which is the most frequently used method in recent years [14].

Although the HPLC‐UV/MS method is not so widely used for the determination of antibiotics which belong to the same group of drugs, its lower price compared to the triple quadrupole instrument makes it, a dynamic analytical method in a routine analysis of drugs. As it is known, the LC‐MS/MS method provides higher sensitivity and selectivity compared to the specifically developed method, however, its use is not preferred in clinical practice because the instrumentation is very expensive and well‐trained personnel is required. A comparison of two analytical systems by the same manufacturing company showed that the HPLC‐PDA/MS system is considered a cost‐effective method, since the cost of the analysis is mainly due to the price of the instrument and not from consumables (e.g., solvents and vials).

## 5 CONCLUDING REMARKS

For the simultaneous determination of VANC and TEIC in urine, an analytical protocol using HPLC‐PDA coupled with an MS system was developed and validated, showing good linearity and high precision. The validation results were excellent and were within the set specifications according to EMA. It is significant to be mentioned that the short analysis time of the developed method is comparable to the other studies using UHPLC‐MS/MS. Moreover, the HPLC‐PDA/MS system is a cost‐effective analytical method, in contrast with the higher price of the UHPLC–MS/MS system. The LOD and LOQ values of VANC were 12–13 times lower than the values of TEIC, indicating that the developed method can be used for the determination of a lower concentration of VANC than TEIC. Nevertheless, the developed analytical method is very sensitive for both glycopeptides in urine. Moreover, as the data shows, the PDA method was more sensitive and less selective than the MS method. For this reason, the combination of the two detection methods provided more reliable results for the analysis of the two glycopeptides in urine. It is significant to be mentioned, that these LOD and LOQ values are in the same order of magnitude or lower compared to the bibliography, showing the sensitivity of the developed method.

The usage of the HPLC‐PDA/MS system requires less effort for the operation of the instrument and less handling of data than more sophisticated instruments such as UHPLC‐MS/MS. These advantages give the opportunity to the scientists to use this developed method for accurate control of analysis of real samples such as biological fluids. Furthermore, the usage of urine samples will facilitate the conduct of clinical pharmacokinetic studies. In future work, the developed analytical protocol will be performed for the analysis of VANC and TEIC in samples of urine from patients, demonstrating its ability to be used in clinical cases.

## CONFLICT OF INTEREST

The authors have declared no conflict of interest.

## Supporting information



Figure S1. Chromatogram of a standard sample of a mixture of vancomycin and teicoplanin at a concentration of 10 mg/L using PDA data.

## Data Availability

The data that support the findings of this study are available in the supplementary material of this article.
