# Supplementary material for: A novel sensitive analytical method for the simultaneous analysis of vancomycin and teicoplanin in human urine via single high‐performance liquid chromatography coupled with photodiode array and mass spectrometry in series
Source: J Sep Sci. 2022 May 20;45(14):2566–81. doi: 10.1002/jssc.202200002 (PMC9543914; doi:10.1002/jssc.202200002)
Supplement: Supplementary file 1 — Figure S1. Chromatogram of a standard sample of a mixture of vancomycin and teicoplanin at a concentration of 10 mg/L using PDA data. [file JSSC-45-2566-s001.docx]

**Supplementary Material**

**PDA**

**TEIC**

**VANC**

**Figure S1.** Chromatogram of a standard sample of a mixture of vancomycin and teicoplanin at concentration of 10 mg/L using PDA data.
